# Efficacy of Naoxueshu in acute spontaneous intracerebral hemorrhage: a multicenter observational study

**DOI:** 10.1007/s10072-021-05582-8

**Published:** 2021-09-16

**Authors:** Juexian Song, Yuting Nie, Xinzuo Qin, Pingping Wang, Huiqiang Lu, Li Gao

**Affiliations:** 1 Department of Neurology, Xuanwu Hospital, Capital Medical University, Beijing, 100053 China; 2 The Key Laboratory of Development Biology, College of Life Sciences, Jinggangshan University, Ji’an, China

**Keywords:** Intracerebral hemorrhage, Hematoma, Traditional Chinese medicine, Naoxueshu, SICH

## Abstract

**Objective:**

To evaluate the efficacy and safety outcome and related risk factors of Naoxueshu in the treatment of acute SICH.

**Methods:**

Two hundred twenty patients were enrolled in this study. Diagnosis of SICH was based on neuroimaging. All the patients received regular treatment and Naoxueshu oral liquid 10 ml 3 times a day for 14 consecutive days. Surgical intervention was conducted as needed. Efficacy and safety outcomes were evaluated.

**Results:**

Hematoma volume decreased significantly 7 days after Naoxueshu treatment (from 27.3 ± 20.0 to 15.1 ± 15.1 ml, *P* < 0.0001), and it decreased further in 14-day result (6.9 ± 10.4 ml, *P* < 0.0001). Patients’ neurological function was improved remarkably with NIHSS scores from baseline 13 points to 7-day 7 points (*P* < 0.0001) and 14-day 4 points (*P* < 0.0001). Cerebral edema was relieved only 14 days after Naoxueshu treatment (from 3 to 2 points, *P* < 0.0001). No clinically significant change was found in 7-day and 14-day safety results. Female sex was related independently to large 7-day hematoma volume and worse 7-day NIHSS score while it would not affect patients’ 14-day outcomes. Rare cause of SICH (*B* = 17.4, *P* = 0.009) alone was related to large 14-day hematoma volume. Worse baseline NIHSS score (*B* = 0.3, *P* = 0.003) and early use of Naoxueshu (*B* = 2.9, *P* = 0.005) were related to worse 7-day and14-day neurological function.

**Conclusion:**

Naoxueshu oral liquid could relieve hematoma volume and cerebral edema safely; meanwhile, it could improve patients’ neurological function. Sex, cause of SICH, and time from onset to receive Naoxueshu should be taken into consideration in the treatment of SICH.

## Introduction

Intracerebral hemorrhage (ICH) accounts for approximately 10% of all strokes [1]. Spontaneous intracerebral hematoma (SICH) is defined as nontraumatic bleeding into the brain parenchyma with abrupt onset of severe headache, altered level of consciousness, and/or focal neurologic deficit [2]. On neuroimaging, we can find focal collection of blood within the parenchyma. SICH is characterized by high mortality and morbidity rate that 30-day mortality has been reported to be approximately 40–50% [3, 4]. SICH has become one of the heavy burdens to the society and the family. However, until today, we have limited treatments for SICH because the mechanism of SICH is not clear. According to the latest AHA/ASA guidelines, reversal of coagulopathy and control of hypertension remain the main therapy of SICH [5].

Finding out an effective neuroprotective drug has been on the schedule of neurologists around the world for many years. Unfortunately, only limited success has been reported in preclinical and clinical trials evaluating numerous compounds [6]. On the other hand, traditional Chinese medicine (TCM) is very popular in all developing countries. According to a 2003 WHO report, it accounts for 30 to 50% of the total medicinal consumption in China [7]. TCM has been used for the treatment of stroke for thousands of years in China. Naoxueshu oral liquid, a TCM patent drug including leech, astragalus root, Rhizome Chuanxiong calamus, and Achyranthes, is designed on the concept of TCM theory that “Qi is the commander of blood; blood is the mother of Qi” [8]. Many small sample studies have demonstrated that Naoxueshu can replenish Qi, activate blood circulation, and remove blood stasis, thus relieving symptoms of SICH [9–13]. The aim of this study was to evaluate the efficacy of Naoxueshu in the treatment of acute SICH in a relatively large-scale population in China. Moreover, we analyzed risk factors that could affect patients’ outcomes when they took Naoxueshu into consideration.

## Material and methods

We prospectively enrolled the patients diagnosed with SICH between December 2016 and August 2019 in 6 hospitals in Beijing, China. Diagnosis of SICH was according to the 2015 AHA/ASA SICH guidelines [5], and was confirmed by magnetic resonance imaging (MRI) or computed tomography (CT). The Ethics Committee of Xuanwu Hospital of Capital Medical University approved this study. Data of all patients were prospectively collected and retrospectively analyzed. The procedures were performed with the patient hospitalized and the written consent of the patients was obtained before the procedure.

### Intervention

After the admission, patients were randomized to Naoxueshu group and Control group. This was an addition on design study. All the patients in two groups received standard of care which include 20% mannitol of dehydration treatment, general monitoring and nursing care, blood pressure control, and supportive therapy. Naoxueshu oral liquid 10 ml 3 times a day was given to the patients for 14 consecutive days in Naoxueshu group. Surgery including craniotomy, decompressive craniectomy, and minimally invasive surgical evacuation was conducted if deteriorating vital signs or neurological function was found in both groups.

### Main outcomes

Efficacy outcomes were hematoma volumes, National Institutes of Health Stroke Scale (NIHSS) scores, and cerebral edema scores of 7 days and 14 days after Naoxueshu treatment. Safety outcomes were 7-day and 14-day changes in blood routine examination, coagulation function, liver function, and renal function. The NIHSS rater was an independent investigator who was blinded of our study in each site. Hematoma volume (ml) = π/6 × length (cm) × width (cm) × high (cm) [14], and it was determined by CT scanning (Siemens 64-slice CT machine, section thickness, 5 mm; gap, 5 mm; pitch, 1; tube current, 304 mA; and voltage, 120 kV).

The CT results were evaluated by an independent radiologist who was blinded in our study with the following protocol, cerebral edema severity was evaluated by a 4-grade scale: (1) No cerebral edema; (2) formation of perihematoma edema zone; (3) manifestation of grade 2 plus ventricle compression; (4) midline shift in addition to manifestation of grade 2 and grade 3.

### Data analysis

All statistical analyses were conducted by SPSS 19.0 (SPSS Inc., Chicago, IL, USA). Continuous variables were presented as means ± standard deviations (SD) if they were normally distributed; if not they were presented as median (interquartile range). The category variables were presented as numbers and frequencies. Outcome comparison was done by repeated measures ANOVAs and all the results of repeated measures ANOVAs were corrected with the Greenhouse–Geisser correction. Post hoc Bonferroni’s test was performed for pairwise comparisons. Risk factors related to efficacy outcomes were analyzed by linear regression. A *P* value less than 0.05 was considered statistically significant.

## Results

### Baseline characteristics

The baseline characteristics of the patients are shown in Table 1. Between December 2016 and August 2019, 220 patients were enrolled in this study. SICH patients were 55.8 ± 11.9 years with male predominant. Vital signs were normal except that both systolic pressure (159.3 ± 25.7 mmHg) and diastolic pressure (96.5 ± 18.1 mmHg) were elevated. Among them, 69.5% were hypertensive ICH, and 27.7% were caused by intracranial vessel malformation. Average time from SICH symptom onset to receive Naoxueshu oral liquid was 1.7 ± 0.8 days.

**Table 1:** Baseline characteristics of the patients

**Characteristics**	
Age (years)	55.8 ± 11.9
Male sex	143 (65.0%)
Heart rate (*n*/min, median, IQR)	80 (71–87)
Respiratory rate (*n*/min, median, IQR)	19 (18–20)
Temperature (℃)	36.7 ± 0.5
Systolic pressure (mmHg)	159.3 ± 25.7
Diastolic pressure (mmHg)	96.5 ± 18.1
Cause of SICH	
Hypertension	153 (69.6%)
Intracranial vessel malformation	61 (27.7%)
Others	6 (2.7%)
Time from onset to receive NXS (days)	1.7 ± 0.8

Notes: *IQR*, interquartile range; *SICH*, spontaneous intracerebral hemorrhage; *NIHSS*, National Institutes of Health Stroke Scale; *NXS*, Naoxueshu oral liquid

### Outcomes comparison

Efficacy outcomes are showed in Table 2. Hematoma volume decreased significantly 7 days after Naoxueshu treatment (from 27.3 ± 20.0 to 15.1 ± 15.1 ml, *P* < 0.0001), and it decreased further in 14-day result (6.9 ± 10.4 ml, *P* < 0.0001). Patients’ neurological function was improved remarkably with NIHSS scores from baseline 13 points to 7-day 7 points (*P* < 0.0001) and 14-day 4 points (*P* < 0.0001). Cerebral edema did not change a lot after 7-day Naoxueshu treatment but it was relieved significantly 14 days after receiving treatments (from 3 to 2 points, *P* < 0.0001).

**Table 2:** Efficacy outcome measures of the patients

Outcomes	Baseline	7-day	14-day	*P* value
Hematoma volume (ml)	27.3 ± 20.0	15.1 ± 15.1	6.9 ± 10.4	< 0.0001
NIHSS score (median, IQR)	13 (4–20)	7 (2–13)	4 (0–10)	< 0.0001
Cerebral edema score (median, IQR)	3 (2–3)	2 (2–3)	2 (1–2)	< 0.0001

Notes: *IQR*, interquartile range; *NIHSS*, National Institutes of Health Stroke Scale

Safety outcomes are showed in Table 3. Seven days after Naoxueshu and other treatment, patients’ thrombin time (TT) decreased by 0.6 s (95% CI: − 1.2 ~  − 0.3, *P* = 0.036), fibrinogen (Fib) increased by 0.8 g/l (95% CI: 0.4 ~ 1.1, *P* < 0.0001), and alanine aminotransferase (ALT) increased by 22.2 U/l (95% CI: 7.0 ~ 37.5, *P* = 0.002). These changes were not significant in 14-day result. On the other hand, hemoglobin (Hgb) decreased by 5.7 g/l (95% CI: − 10.9 ~  − 0.5, *P* = 0.028) and platelet (PLT) increased by 41.0 × 10^9^/l (95% CI: 13.7 ~ 68.3, *P* = 0.002) 14 days after treatment.

**Table 3:** Safety outcome measures of the patients

Outcomes	Baseline	7-day	14-day	*P* value
WBC (× 10^9^/l)	10.5 ± 4.5	9.2 ± 3.0	8.0 ± 3.2	0.013
Hgb (g/l)	137.0 ± 19.2	130.9 ± 22.3	130.1 ± 18.5	0.002
PLT (× 10^9^/l)	200.4 ± 73.7	228.3 ± 76.6	244.2 ± 83.9	< 0.0001
APTT (s)	29.2 ± 6.6	29.2 ± 6.5	30.3 ± 5.9	0.269
PT (s)	12.5 ± 2.4	12.3 ± 2.0	12.2 ± 1.8	0.211
TT (s)	16.7 ± 3.0	16.1 ± 2.9	16.0 ± 2.7	0.012
Fib (g/l)	3.1 ± 1.1	3.8 ± 1.6	3.7 ± 1.4	< 0.0001
ALT (U/l)	26.2 ± 15.9	43.7 ± 39.2	40.4 ± 39.9	< 0.0001
AST (U/l)	28.1 ± 16.2	39.8 ± 58.6	35.5 ± 37.6	0.034
TBIL (μmol/l)	17.9 ± 8.5	16.7 ± 11.5	13.5 ± 7.8	0.018
Cr (μmol/l)	64.2 ± 24.6	62.1 ± 21.2	63.1 ± 32.4	0.570
BUN (mmol/l)	6.3 ± 7.3	8.8 ± 10.5	8.7 ± 14.7	0.902

Notes: *WBC*, white blood cell; *Hgb*, hemoglobin; *PLT*, platelet; *APTT*, activated partial thromboplastin time; *PT*, prothrombin time; *TT*, thrombin time; *Fib*, fibrinogen; *ALT*, alanine aminotransferase; *AST*, aspartate aminotransferase; *TBIL*, total bilirubin; *Cr*, creatinine; *BUN*, blood urea nitrogen

### Linear regression of risk factors

#### Factors related to hematoma volume

Male sex was related independently to small 7-day hematoma volume (*B* =  − 8.5, 95% CI: − 15.5 ~  − 1.5, *P* = 0.019, Table 4). If SICH was not caused by hypertension or intracranial vessel malformation, it was more likely linking to a large 7-day hematoma volume (*B* = 20.4, 95% CI: 2.0 ~ 38.8, *P* = 0.031, Table 4). Patients without craniocerebral surgery seemed to have higher risk of large 7-day hematoma volume (*B* = 8.6, 95% CI: 0.2 ~ 17.0, *P* = 0.045, Table 4). Not surprisingly, large baseline hematoma volume was related to large 7-day hematoma volume (*B* = 0.3, 95% CI: 0.03 ~ 0.6, *P* = 0.030, Table 4). However, only rare cause of SICH (not hypertensive or caused by intracranial vessel malformation) was related to large 14-day hematoma volume (*B* = 17.4, 95% CI: 4.6 ~ 30.1, *P* = 0.009, Table 4).

**Table 4:** Factors related to hematoma volume

Factors	7-day hematoma volume		14-day hematoma volume	
	***B* (95% CI)**	***P* value**	***B* (95% CI)**	***P* value**
Age	0.01 (− 0.3 ~ 0.3)	0.963	0.04 (− 0.2 ~ 0.2)	0.673
Male sex	− 8.5 (− 15.5 ~ − 1.5)	0.019	− 1.7 (− 6.6 ~ 3.1)	0.468
Heart rate	0.05 (− 0.2 ~ 0.2)	0.623	− 0.04 (− 0.2 ~ 0.1)	0.580
Respiratory rate	0.4 (− 1.0 ~ 1.8)	0.572	0.7 (− 0.3 ~ 1.6)	0.167
Temperature	− 2.4 (− 7.0 ~ 2.2)	0.301	− 1.1 (− 4.3 ~ 2.1)	0.486
Systolic pressure	0.07 (− 0.06 ~ 0.2)	0.270	0.04 (− 0.05 ~ 0.1)	0.370
Diastolic pressure	− 0.08 (− 0.3 ~ 0.1)	0.422	0.02 (− 0.1 ~ 0.2)	0.765
Cause of SICH				
Hypertension	/	/	/	/
Intracranial vessel malformation	0.1 (− 7.4 ~ 7.6)	0.970	2.9 (− 2.3 ~ 8.1)	0.262
Others	20.4 (2.0 ~ 38.8)	0.031	17.4 (4.6 ~ 30.1)	0.009
Baseline hematoma volume	0.3 (0.03 ~ 0.6)	0.030	0.09 (− 0.1 ~ 0.3)	0.382
Baseline NIHSS score	0.002 (− 0.4 ~ 0.4)	0.991	− 0.05 (− 0.2 ~ 0.3)	0.695
Baseline cerebral edema score	− 1.1 (− 4.1 ~ 1.9)	0.475	− 1.3 (− 3.4 ~ 0.8)	0.209
Surgery				
Yes	/	/	/	/
No	8.6 (0.2 ~ 17.0)	0.045	3.0 (− 2.8 ~ 8.8)	0.297
Time from onset to receive NXS	− 0.1 (− 3.7 ~ − 3.5)	0.960	− 0.6 (− 3.1 ~ 1.9)	0.652

Notes: *SICH*, spontaneous intracerebral hemorrhage; *NIHSS*, National Institutes of Health Stroke Scale; *NXS*, Naoxueshu oral liquid

#### Factors related to neurological function

Again, male sex was related independently to better 7-day NIHSS score (*B* =  − 4.4, 95% CI: − 7.6 ~  − 1.25, *P* = 0.009, Table 5). Patients’ respiratory rate (*B* = 0.7, 95% CI: 0.1 ~ 1.3, *P* = 0.032) and body temperature (*B* =  − 2.2, 95% CI: − 4.4 ~  − 0.1, *P* = 0.039, Table 5) at the time when SICH symptom onset could also affect their 7-day NIHSS score. Apparently, worse baseline NIHSS score was related to worse 7-day NIHSS score (*B* = 0.5, 95% CI: 0.4 ~ 0.7, *P* < 0.0001) and 14-day NIHSS score (*B* = 0.3, 95% CI: 0.1 ~ 0.5, *P* = 0.003, Table 5). Interestingly, immediate use of Naoxueshu seemed to have worse 7-day (*B* =  − 3.4, 95% CI: − 5.1 ~  − 1.7, *P* < 0.0001) and 14-day NIHSS score (*B* =  − 2.9, 95% CI: − 4.9 ~  − 1.0, *P* = 0.005, Table 5).

**Table 5:** Factors related to neurological function

Factors	7-day NIHSS score		14-day NIHSS score	
	***B* (95% CI)**	***P* value**	***B* (95% CI)**	***P* value**
Age	0.02 (− 0.1 ~ 0.2)	0.702	0.02 (− 0.1 ~ 0.2)	0.762
Male sex	− 4.4 (− 7.6 ~ − 1.2)	0.009	− 3.4 (− 7.2 ~ 0.4)	0.079
Heart rate	0.1 (− 0.02 ~ 0.3)	0.103	0.1 (− 0.04 ~ 0.2)	0.229
Respiratory rate	0.7 (0.1 ~ 1.3)	0.032	0.4 (− 0.4 ~ 1.1)	0.331
Temperature	− 2.2 (− 4.4 ~ − 0.1)	0.039	− 2.2 (− 4.8 ~ 0.3)	0.079
Systolic pressure	0.02 (− 0.04 ~ 0.1)	0.471	0.01 (− 0.1 ~ 0.1)	0.776
Diastolic pressure	− 0.04 (− 0.1 ~ 0.1)	0.409	− 0.1 (− 0.2 ~ 0.1)	0.379
Cause of SICH				
Hypertension	/	/	/	/
Intracranial vessel malformation	− 0.06 (− 3.4 ~ 3.5)	0.973	0.7 (− 3.4 ~ 4.8)	0.721
Others	6.8 (− 1.6 ~ 15.3)	0.110	3.6 (− 6.5 ~ 13.7)	0.472
Baseline hematoma volume	0.02 (− 0.1 ~ 0.2)	0.750	− 0.03 (− 0.2 ~ 0.1)	0.721
Baseline NIHSS score	0.5 (0.4 ~ 0.7)	< 0.0001	0.3 (0.1 ~ 0.5)	0.003
Baseline cerebral edema score	− 0.2 (− 1.6 ~ 1.2)	0.774	0.7 (− 1.0 ~ 2.3)	0.406
Surgery				
Yes	/	/	/	/
No	− 1.5 (− 5.3 ~ 2.4)	0.443	− 2.7 (− 7.3 ~ 1.9)	0.235
Time from onset to receive NXS	− 3.4 (− 5.1 ~ − 1.7)	< 0.0001	− 2.9 (− 4.9 ~ − 1.0)	0.005

Notes: *SICH*, spontaneous intracerebral hemorrhage; *NIHSS*, National Institutes of Health Stroke Scale; *NXS*, Naoxueshu oral liquid

No statistically significant risk factor was found that was related to cerebral edema (Table 6).

**Table 6:** Factors related to cerebral edema

Factors	7-day cerebral edema score		14-day cerebral edema score	
	***B* (95% CI)**	***P* value**	***B* (95% CI)**	***P* value**
Age	− 0.01 (− 0.05 ~ 0.03)	0.519	− 0.003 (− 0.04 ~ 0.04)	0.876
Male sex	− 0.2 (− 0.8 ~ 1.1)	0.728	0.2 (− 0.7 ~ 1.2)	0.608
Heart rate	− 0.02 (− 0.05 ~ 0.006)	0.121	− 0.01 (− 0.04 ~ 0.02)	0.358
Respiratory rate	0.08 (− 0.1 ~ 0.3)	0.398	− 0.01 (− 0.2 ~ 0.2)	0.891
Temperature	0.3 (− 0.4 ~ 0.9)	0.406	− 0.1 (− 0.8 ~ 0.5)	0.637
Systolic pressure	0.0001 (− 0.02 ~ 0.02)	0.959	− 0.005 (− 0.02 ~ 0.01)	0.581
Diastolic pressure	0.01 (− 0.01 ~ 0.05)	0.277	0.02 (− 0.01 ~ 0.05)	0.160
Cause of SICH				
Hypertension	/	/	/	/
Intracranial vessel malformation	0.09 (− 0.9 ~ 1.1)	0.856	− 0.5 (− 1.5 ~ 0.6)	0.366
Others	1.1 (− 1.4 ~ 3.7)	0.367	1.5 (− 1.1 ~ 4.0)	0.246
Baseline hematoma volume	− 0.01 (− 0.05 ~ 0.03)	0.739	− 0.003 (− 0.04 ~ 0.04)	0.885
Baseline NIHSS score	− 0.01 (− 0.06 ~ 0.05)	0.847	0.007 (− 0.04 ~ 0.06)	0.779
Baseline cerebral edema score	0.3 (− 0.1 ~ 0.8)	0.109	0.3 (− 0.1 ~ 0.7)	0.139
Surgery				
Yes	/	/	/	/
No	− 0.2 (− 1.4 ~ 1.0)	0.737	− 0.4 (− 1.5 ~ 0.8)	0.508
Time from onset to receive NXS	0.1 (− 0.4 ~ 0.6)	0.590	− 0.06 (− 0.6 ~ 0.4)	0.802

Notes: *SICH*, spontaneous intracerebral hemorrhage; *NIHSS*, National Institutes of Health Stroke Scale; *NXS*, Naoxueshu oral liquid

## Discussion

The results of our study show that Naoxueshu oral liquid is an effective and safe treatment in reducing hematoma volume and cerebral edema in SICH.

Use of Naoxueshu oral liquid in stroke treatment can be dated back to the year 2000. Nearly two decades later, in 2017, a systematic review and meta-analysis of 14 studies (1606 patients) was conducted and revealed the efficacy of Naoxueshu in SICH [15]. However, high heterogeneity was found in some of their results, and the authors explained that the reasons might be related to the number of samples, the quality of the literature, the subject of study, and the duration of treatment. In our study, we controlled Naoxueshu taking time (14 days) in a relatively large-scale population (220 subjects) and confirmed the result that Naoxueshu could obviously promote the absorption of intracranial hematoma; promote the recovery of neurological function, effectively reduce brain edema; and improve the prognosis of patients. Cerebral edema after brain injury was considered dangerous because of the following high intracranial pressure and cerebral hernia. Medical treatment for cerebral edema was restricted to osmolar agents such as mannitol and hypertonic saline, and corticosteroids [16]. However, they had limited efficacy with a relatively high potential of risk. Numerous neuroprotective agents had been tested in preclinical models or small clinical trials in patients. To our knowledge, the results were disappointing. Studies demonstrated that components in Naoxueshu oral liquid could reduce inflammatory response and protect blood–brain barrier (BBB) from disruption [17, 18]. Our study found that Naoxueshu treatment could gradually relieve cerebral edema after SICH (from baseline 3 points to 14-day 2 points, *P* < 0.0001) and it might have potential ability to prevent late cerebral edema.

Although TCM is very popular in Asian countries, safety is the main concern when it is practiced in western countries. Because the active components of TCM often have not been specified and measured precisely, TCM may have direct toxic to the liver or kidney. Moreover, allergic reactions and interactions with other medicines in TCM use can also be seen in some reports. Our study recorded changes in patients’ blood routine examination, coagulation function, liver function, and renal function. Gradually increase in PLT (from baseline 200.4 × 109/l to 14-day 244.2 × 109/l, *P* = 0.002), which indicated recovery from bleeding, was found. In addition, we also found the level of fib and ALT were elevated 7 days after Naoxueshu treatment. This might be caused by the release of thrombin combined with fibrin secondarily after cerebral hemorrhage, in order to block the ruptured blood vessels and prevent rebleeding. The elevated thrombin and fibrin would cause the increase of ALT and AST levels. This increase was short term and it began to decrease in 14-day results. Changes of Hgb might be related to loss of blood and changes of TT were considered not clinically significant.

Up till now, over 10 clinical grading scores for ICH have been developed in order to assist its prognostication [19–26]. The ICH score revealed that factors independently associated with 30-day mortality were Glasgow Coma Scale (GCS) score, age ≥ 80 years, infratentorial origin of ICH, ICH volume, and presence of intraventricular hemorrhage [19]. The ICH-GS confirmed the predictors but with different cutoffs and scoring to improve the prognostic power of the predictors [23]. Another important grading scale for ICH prognosis prediction was the FUNC score, which could accurately identify patients with low chance of functional neurologic recovery at discharge. Related predictive factors were age, GCS, ICH location, volume, and pre-ICH cognitive impairment [24]. In our study, besides high baseline NIHSS score, female patients with high respiratory rate and low body temperature might lead to unfavorable 7-day neurologic recovery. However, these factors did not have an impact on 14-day neurological function. Importantly, immediate use of Naoxueshu was associated to worse 7-day and 14-day NIHSS score. There are many factors that affect the deterioration of neurological function in the early stage of cerebral hemorrhage, such as fluctuations in blood pressure, the influence of surgery, and poor control of cerebral edema, so it seems the early neurological function deterioration of cerebral hemorrhage, but it still needs to be comprehensively considered, and pay more attention to the final improvement of neurological function. However, we still consider a relatively later use of Naoxueshu after SICH onset in practice.

Hypertension and intracranial vessel malformation were two major causes of SICH. According to our study, 69.5% of all the SICH patients were hypertensive, which was higher than the results reported by Finland [27] and the USA [28]. Intracranial vessel malformation led to 27.7% of SICH, and it was in accordance with the result from another study in Chinese population [29]. Other rare cause of SICH could be internal disease, hematological disease, brain tumor, and some unidentified causes. Although this rare cause only accounted for 2.7% of all SICH, it was independently related to 7-day (*B* = 20.4, *P* = 0.031) and 14-day hematoma volume (*B* = 17.4, *P* = 0.009). Moreover, surgery relieved hematoma volume temporarily because 14-day result was not statistically significant. That supported the fact that efficacy of surgical removal of the hemorrhage was still “unclear” [30].

Besides the risk factors discussed above, studies also revealed that homogeneous and regularly shaped small ICH, or benign ICH, and intraventricular hemorrhage (IVH) growth defined as any newly occurring intraventricular bleeding in patients without baseline IVH or an increase in IVH volume ≥ 1 ml in patients with initial IVH on follow-up CT scan within 36 h after the baseline CT scan were useful risk prediction predictors for outcome in ICH patients [31, 32]. Moreover, although in our study patients’ blood pressure was not a risk factor related to hematoma volume or cerebral edema, there was an opinion that ultra-early blood pressure reduction within 2 h after onset of symptoms could be an optimal treatment strategy for ICH [33].

The main limitation of our study was that we used a 4-grade scale to evaluate cerebral edema. It was easy to operate in all the 6 hospitals but it was not so accurate. This might affect the results of liner regression in cerebral edema analysis. Second, there is no control group in this observational study, so natural course of hematoma absorption with the increase of time after ICH onset could not be evaluated and analyzed. For the BMI and blood lipid parameters which were also very important to evaluate the outcomes, we did not collect previously, which may have some impact on our conclusion.

## Conclusion

Naoxueshu oral liquid could relieve hematoma volume and cerebral edema safely; meanwhile it could improve patients’ neurological function. Sex, cause of SICH, and time from onset to receive Naoxueshu should be taken into consideration in the treatment of SICH. Female patients with SICH that was not caused by hypertension or intracranial vessel malformation should be treated carefully with relatively later use of Naoxueshu oral liquid.

## Data Availability

The original data will be available when contacting the correspondence author.

## References

[1] Woo D, Broderick JP (2002). Spontaneous intracerebral hemorrhage: epidemiology and clinical presentation. Neurosurg Clin N Am.

[2] National Institute of Neurological Disorders and Stroke (1990). Classification of cerebrovascular diseases III. Stroke.

[3] Broderick JP, Brott TG, Duldner JE (1993). Volume of intracerebral hemorrhage a powerful and easy-to-use predictor of 30-day mortality. Stroke.

[4] Hemphill JC, Bonovich DC, Besmertis L (2001). The ICH score: a simple, reliable grading scale for intracerebral hemorrhage. Stroke.

[5] Hemphill JC, Greenberg SM, Anderson CS (2015). Guidelines for the management of spontaneous intracerebral hemorrhage: a guideline for healthcare professionals from the American Heart Association/American Stroke Association. Stroke.

[6] Chamorro Á, Dirnagl U, Urra X, Planas AM (2016). Neuroprotection in acute stroke: targeting excitotoxicity, oxidative and nitrosative stress, and inflammation. Lancet Neurol.

[7] World Health Organization. World Health Organization Fact Sheet. Revised May 2003 ed. Geneva: World Health Organization; 2003.

[8] Chen C, Yuan LX, Zhang GM (2014). Clinical application and experimental research progress of nao-xue-shu oral liquid in cerebrovascular disease. Chinese Journal of Integrated Traditional Chinese and Western Medicine.

[9] Wang XG, Zhao XQ (2015). Paired study on the treatment of acute cerebral hemorrhage with nao-xue-shu oral liquid. Chinese Journal of Integrative Medicine on Cardio-/Cerebrovascular Disease.

[10] Chen SJ, Wang HY, Zuo Y (2016). Clinical observation on the treatment of secondary brain damage after intracerebral hemorrhage by Nao-Xue-Shu oral liquid. Chin J Integr Med.

[11] Miao WC, Yan R (2014). Clinical observation on the treatment of hypertensive cerebral hemorrhage with naoxueshu oral liquid. Chinese Journal of Practical Nervous Disease.

[12] Wang H, Niu XZ, Su TS (2016). Clinical observation on the treatment of cerebral hemorrhage in convalescent stage with acupuncture combined with naoxueshu oral liquid. World Latest Medicine Information.

[13] Li SH, Han XH, Jiang GY (2014). Clinical observation on the treatment of hypertensive cerebral hemorrhage with minimally invasive surgery of intracranial hematoma combined with naoxueshu oral liquid. Chinese Journal of Practical Nervous Diseases.

[14] Song J, Lyu Y, Wang P (2018). Treatment of Naoxueshu promotes improvement of hematoma absorption and neurological function in acute intracerebral hemorrhage patients. Front Physiol.

[15] Wu L, Li Y, Wang X (2017). A systematic review and meta-analysis on the treatment of cerebral hemorrhage with NaoXueShu oral liquid. Biomed Res Int.

[16] Kim H, Edwards NJ, Choi HA (2016). Treatment strategies to attenuate perihematomal edema in patients with intracerebral hemorrhage. World Neurosurg.

[17] Dong H, Ren JX, Wang JJ (2016). Chinese medicinal leech: ethnopharmacology, phytochemistry, and pharmacological activities. Evid Based Complement Alternat Med.

[18] Li KM, Zhang G, Wu JB (2007). Overview of pharmacological research of leech. Tradit Chin Med Res.

[19] Hemphill JC, Bonovich DC, Besmertis L (2011). The ICH score: a simple, reliable grading scale for intracerebral hemorrhage. Stroke.

[20] Cheung RT, Zou LY (2003). Use of the original, modified, or new intracerebral hemorrhage score to predict mortality and morbidity after intracerebral hemorrhage. Stroke.

[21] Godoy DA, Pinero G, Di Napoli M (2006). Predicting mortality in spontaneous intracerebral hemorrhage: can modification to original score improve the prediction?. Stroke.

[22] Weimar C, Benemann J, Diener HC (2006). Development and validation of the Essen intracerebral haemorrhage score. J Neurol Neurosurg Psychiatry.

[23] Ruiz-Sandoval JL, Chiquete E, Romero-Varguss S (2007). Grading scale for prediction of outcome in primary intracerebral hemorrhages. Stroke.

[24] Rost NS, Smith EE, Chang Y (2008). Prediction of functional outcome in patients with primary intracerebral hemorrhage: the FUNC score. Stroke.

[25] Cho DY, Chen CC, Lee WY (2008). A new modified intracerebral hemorrhage score for treatment decision in basal ganglia hemorrhage: a randomized trial. Crit Care Med.

[26] Chaung YC, Chen YM, Peng SK, Peng SY (2009). Risk stratification for predicting 30-day mortality of intracerebral hemorrhage. Int J Qual Health Care.

[27] Fogelholm R, Murros K (1993). Cigarette smoking and risk of primary intracerebral haemorrhage. A population-based case-control study. Acta Neurol Scand.

[28] Feldmann E, Broderick JP, Kernan WN (2005). Major risk factors for intracerebral hemorrhage in the young are modifiable. Stroke.

[29] Luo J, Wang YH, Zhong XN (2008). Analysis of etiological factor, risk factor and prognosis of 158 young patients with intracerebral hemorrhage. Modern Preventive Medicine.

[30] Manners J, Steinberg A, Shutter L (2017). Early management of acute cerebrovascular accident. Curr Opin Crit Care.

[31] Li Q, Yang WS, Shen YQ (2019). Benign intracerebral hemorrhage: a population at low risk for hematoma growth and poor outcome. J Am Heart Assoc.

[32] Li Q, Li R, Zhao LB (2020). Intraventricular hemorrhage growth: definition, prevalence and association with hematoma expansion and prognosis. Neurocrit Care.

[33] Li Q, Warren AD, Qureshi AI (2020). Ultra-early blood pressure reduction attenuates hematoma growth and improves outcome in intracerebral hemorrhage. Ann Neurol.

